# Economic evaluation of interventions designed to reduce *Clostridium difficile* infection

**DOI:** 10.1371/journal.pone.0190093

**Published:** 2018-01-03

**Authors:** David Brain, Laith Yakob, Adrian Barnett, Thomas Riley, Archie Clements, Kate Halton, Nicholas Graves

**Affiliations:** 1 Queensland University of Technology, Institute of Health and Biomedical Innovation, Brisbane, Queensland, Australia; 2 London School of Hygiene and Tropical Medicine, Faculty of Infectious and Tropical Diseases, London, United Kingdom; 3 University of Western Australia, Pathology and Laboratory Medicine, Perth, Western Australia, Australia; 4 Australian National University, Research School of Population Health, Canberra, Australian Capital Territory, Australia; Cornell University, UNITED STATES

## Abstract

**Introduction:**

Healthcare decision-makers are increasingly expected to balance increasing demand for health services with a finite budget. The role of economic evaluation in healthcare is increasing and this research provides decision-makers with new information about the management of *Clostridium difficile* infection, from an economic perspective.

**Methods:**

A model-based economic evaluation was undertaken to identify the most cost-effective healthcare intervention relating to the reduction of *Clostridium difficile* transmission. Efficacy evidence was synthesised from the literature and was used to inform the effectiveness of both bundled approaches and stand-alone interventions, where appropriate intervention combinations were coupled together. Changes in health outcomes were estimated by combining information about intervention effectiveness and its subsequent impact on quality of life.

**Results:**

A bundled approach of improving hand hygiene and environmental cleaning produces the best combination of increased health benefits and cost-savings. It has the highest mean net monetary benefit when compared to all other interventions. This intervention remains the optimal decision under different clinical circumstances, such as when mortality rate and patient length of stay are increased. Bundled interventions offered the best opportunity for health improvements.

**Conclusion:**

These findings provide healthcare decision-makers with novel information about the allocation of scarce resources relating to *Clostridium difficile*. If investments are not made in interventions that clearly yield gains in health outcomes, the allocation and use of scarce healthcare resources is inappropriate and improvements in health outcomes will be forgone.

## Introduction

*Clostridium difficile (C*. *difficile)* is the most common cause of hospital-acquired infectious diarrhoea and represents a significant challenge for health services [1]. It is primarily transmitted via the fecal-oral pathway, through ingestion of bacterial spores [2]. The majority of cases follow the use of antibiotics, which disturb the resident gut microflora [2]. Other risk factors include advanced age, frequent admission to hospital and comorbidity [3]. Infection can cause a range of clinical symptoms, including severe diarrhoea, pseudomembranous colitis, and in extreme cases, death [4]. It is the most common cause of infectious, hospital-acquired diarrhoea and is reported to be responsible for 15–25% of antibiotic associated diarrhoea [2; 5] Following the spread of hypervirulent strains, most notably ribotype 027, an increase in severe cases has been documented in studies from the UK, Europe and North America [6]. Recurrent infection, where patients are treated for their initial infection then suffer either a relapse or reinfection, is a common feature of the disease [7]. Recurrence rates have been reported to be 25% of all cases (range 6–42%), increasing patient vulnerability and debilitation while incurring economic costs through increased patient length of stay and hospitilisation [8]. Information on the attributable cost of *C*. *difficile* infection (CDI) varies, with estimates of primary infection costs ranging from USD $3,400–$16,300 per case [9] and recurrent infection between USD $13,700–$18,000 [10]. CDI-attributable mortality has also increased, with pre-2000 estimates of 1.5% mortality increasing to between 4.5% and 5.7% in recent endemic periods [9].

There are standardised guidelines for controlling CDI [11] however, they are strategy-level, not particularly prescriptive and tend to be reactive. There are many infection control intervention options available to reduce transmission and most hospitals employ a mixed approach that combines antimicrobial stewardship, hand hygiene, environmental cleaning and fecal bacteriotherapy. There is paucity of economic evidence to support existing practice, with the majority of guidelines built solely on clinical evidence, with no consideration of the costs and health returns from alternative strategies of infection c0ntrol. Scarce resources for infection prevention should be used first in those programmes that deliver the greatest health returns per dollar invested [12–13]. The aim of this paper is to provide useful evidence for healthcare decision-makers about the cost-effectiveness of competing investments for prevention and for control of CDI.

## Methods

Although conducted for an Australian hospital setting, methods described here can easily be transferred to alternative settings. A hospital system perspective has been adopted for this evaluation, meaning that costs relating to treatment, administration, in-hospital resource use, the monitoring of and treatment of adverse events have been included. Personal costs, such as out-of-pocket medication, transportation, lost income or co-payment for follow-up care, have not been included.

### Model structure

**Fig 1:**
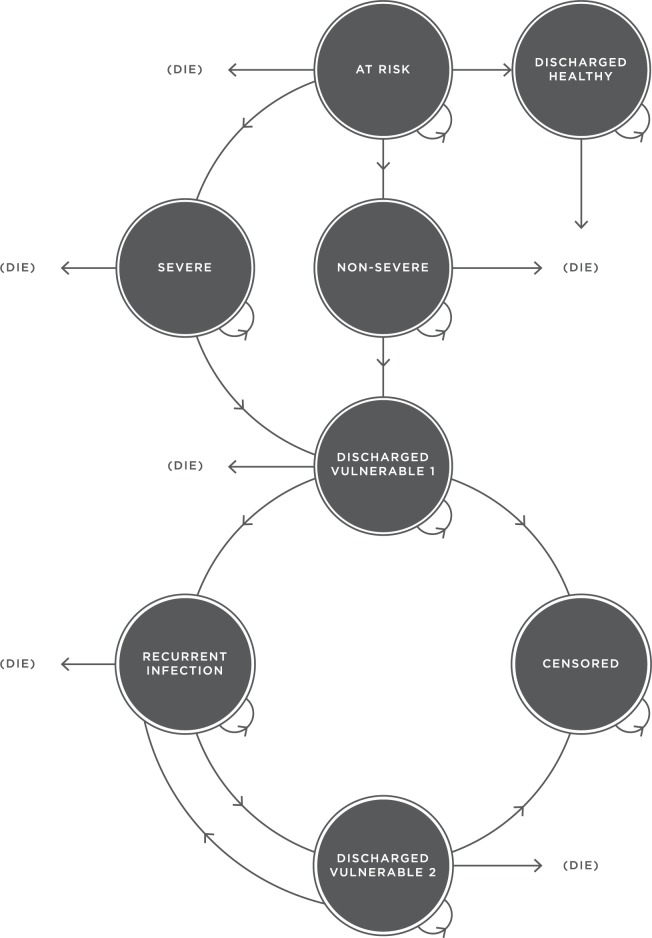
Pictorial representation of the Markov model used to estimate costs and QALYs for patients with *C*. *difficile*.

A decision analytic model was developed to evaluate the cost-effectiveness of interventions that reduce CDI [14]. Given the chronic and repetitive nature of the disease, the model needed to be state-based and able to include recursive events. A Markov model was developed based on the natural history of the disease and data from Australian hospitals. The model depicts the movement of adult patients through numerous health states over time, with each health state having a cost and health utility weight attached to time spent in that state. This structure provides the framework for the evaluation and is used to estimate the costs and health outcomes associated with each intervention. The model (Fig 1) includes two absorbing states–‘death’ and ‘censored’. Patients are censored when their timeline reaches the limit of the available data, which was collected over an 18-month period. For example, we used observed data on those who were discharged from hospital in a vulnerable state and did not return to hospital during the length of available follow-up time, but who may have returned if we had a longer follow-up time. Death can occur from any health state, except censored. This model only applies to healthcare-associated infection and does not include infections that have been imported to the hospital from the community. As such, all patients begin in the health state ‘at-risk’ and represent newly admitted patients. Patients can either remain in the ‘at-risk’ state or transition into one of three states: ‘non-severe infection’, ‘severe infection’ or ‘discharged healthy’. It is assumed that there is no possibility of movement between the two infective states. Patients who suffer an infection ultimately leave the system only either in the state ‘discharged vulnerable 1’ or ‘death’. All patients who have suffered infection are classified as ‘vulnerable’ due to the damage done to the gut’s microbiota and their exposure to antibiotics during treatment. Patients in the state ‘discharged vulnerable 1’ can remain in this state, experience a recurrence and move to ‘recurrent infection’, or enter either absorbing state. Due to a lack of data clarity, recurrent patients could not be categorised according to disease severity, so we assumed that all recurrent infections were severe. Patients who have a recurrent infection are transitioned from their infective category to a vulnerable state—‘discharged vulnerable 2’—or one of the absorbing states. Patients who are ‘discharged vulnerable 2’ but who avoid another infection recurrence or death will eventually transition to ‘censored’. The model structure emerged from consultations with infection control nurses and infectious disease physicians to ensure it resembles reality.

### Interventions included in the evaluation

All strategies were compared with ‘standard care’, which was assumed to be: a current antimicrobial stewardship programme or antimicrobial restriction policy (AMS), a hand hygiene and environmental cleaning programme for the whole hospital, and the capacity to undertake fecal microbiota transplant (FMT). Rates consistent with those previously published were used [15]: the baseline level of antimicrobial exposure in a hospital at any given time was assumed to be 50% of inpatients [16–17]; the average time for gut flora to be properly restored was 90 days post-infection [18]; and the average length of stay for all hospitalised patients was assumed to be 5.8 days [19].

The interventions considered are described in Table 1. A panel of experienced infection control clinicians screened all interventions for clinical plausibility and practicality, with intervention bundles being included to reflect the reality of practice in service delivery [20]. A pragmatic approach was taken when choosing which interventions were included in the final analysis. This was especially important when considering bundled interventions, with only those that were deemed to be realistically plausible included, to minimise the likelihood of overestimating transmission reduction. In addition, some possible bundle combinations, such as AMS and FMT, were deliberately omitted due to their clinical implausibility. This model is a flexible framework for the evaluation of numerous infection control strategies and includes a range of efficacy estimations for each intervention, based on published estimates. The inclusion of a range of efficacy estimates represents modelled changes to *C*. *difficile* transmission rates and shows the range of results that may be achievable under certain local conditions. The research team is happy to share the full model on an open access platform for others to evaluate other strategies, or different strategy efficacy levels that are not explicitly shown in these results and has made all input parameter information available in text and within supporting information files.

**Table 1:** Interventions included in cost-effectiveness analysis.

Intervention (Modelled Change)	Description
**AMS 1** **(Large Reduction)**	An antimicrobial stewardship programme that reduces antibiotic use across the hospital such that patients in the vulnerable category are reduced from 50% to 25% (best reported reduction attributed to AMS programmes)
**AMS 2** **(Moderate Reduction)**	An antimicrobial stewardship programme that reduces antibiotic use across the hospital such that patients in the vulnerable category are reduced from 50% to 40% (average reported reduction attributed to AMS programmes)
**HYG 1** **(Large Reduction)**	A hygiene improvement intervention that has the effect of reducing the transmission rate by half
**HYG 2** **(Moderate Reduction)**	A hygiene improvement intervention that has the effect of reducing the transmission rate by a quarter
**FMT 1** **(Moderate Reduction)**	Expedited gut recovery due to FMT for infected patients (time to recovery halved– 45 days)
**FMT 2** **(Large Reduction)**	Expedited gut recovery due to FMT for infected patients (best reported recovery rate– 10 days)
**FMT 3** **(Conservative Reduction)**	Expedited gut recovery due to FMT for infected patients (worst reported recovery rate– 62 days)
**AMS & HYG** **(Moderate AMS, Large HYG Reduction)**	An antimicrobial stewardship programme and hygiene improvement programme delivered as a bundle (reduction in antibiotic use from 50% to 40% of patients; transmission rate halved due to effectiveness of hygiene improvement programme)
**HYG & FMT 1** **(Moderate FMT, Large HYG Reduction)**	A hygiene improvement programme delivered in conjunction with FMT for recurrently infected patients (gut recovery time halved due to FMT; transmission rate halved due to effectiveness of hygiene improvement programme)
**HYG & FMT 2** **(Large FMT, Large HYG Reduction)**	A hygiene improvement programme delivered in conjunction with FMT for recurrent patients (best reported gut recovery rate due to FMT; transmission rate halved due to effectiveness of hygiene improvement programme)

*Hygiene improvement (HYG)* is a combination of two fundamental infection control strategies–hand hygiene and environmental cleaning. Hand hygiene programmes are part of routine hospital infection control practice and the efficacy of environmental cleaning is presented elsewhere [11;21]. *Antimicrobial stewardship (AMS)* is a common intervention that targets a major risk factor in *C*. *difficile* transmission–the overuse of antibiotics. AMS programmes are designed to encourage more careful and controlled prescribing practices, to improve patient outcomes and limit antibiotic associated disease and resistance [22–23]. The effectiveness of AMS programmes has been the subject of debate, with achievable reduction in antimicrobial use being reported to range from 5% to 60% [24–25]. *Fecal microbiota transplant (FMT)* is where a stool sample from a non-infected donor is transplanted into the infected patient in an attempt to restore their normal gut microbiota. Whilst not commonly thought of as a traditional infection control intervention, there is growing evidence that FMT is particularly effective in treating recurrent infections [26]. Health services can utilise FMT as a transmission-inhibiting intervention as its successful implementation as a therapeutic option will limit in-hospital transmission from recurrent patients.

### Data sources

The model is populated with data from a range of sources (Table 2). The estimation of intervention cost followed a published framework of *identifying*, *measuring and valuing* resources; to record only the expenditure required to produce a health effect [27]. Inventories detailing the resources required to physically run each intervention in a real-world hospital were developed and can be made available by request, enabling this study’s method to be tailored for different health systems around the world. A microcosting approach was taken to value the medical resources required for all interventions in 2015 Australian dollars. Microcosting includes detailing all inputs used in the treatment of a particular patient [28]. Non-medical resources, such as staff time, were estimated using data from the Queensland Department of Health’s human resources department. The estimation of infection-related costs was categorised by *diagnosis costs*, *treatment costs and valuation of bed-days lost to CDI*. Diagnosis costs included all costs incurred by the healthcare system when identifying and confirming the presence of infection. Only the cost of the most common treatment regimens was included in the analysis, with specialty regimens or rarely used drugs deliberately omitted. Treatment regimens for all infected states were informed by the Australasian Society of Infectious Diseases guidelines for treatment and costs were informed by the Australian *Pharmaceutical Benefits Scheme* (PBS) [29]. The value of ICU-bed and ward-bed days was informed by published estimates [30–31] and different methods for valuing bed days in monetary terms was explored in scenario analysis.

**Table 2:** Input variables for the Markov model.

Variable	Fixed Value	Range	Distribution	Reference
**Health Utilities**	**(Daily)**			
At-Risk	0.92	0.84–0.96	Uniform	[34]
Non-Severe	0.82	0.72–0.93	Uniform	[34]
Severe	0.71	0.50–0.72	Uniform	[34]
Discharged Vulnerable 1	0.85	0.75–0.90	Uniform	[35]
Recurrent Infection	0.61	0.50–0.72	Uniform	[35]
Discharged Vulnerable 2	0.80	0.70–0.85	Uniform	[35]
Discharged Healthy	0.88	0.84–0.92	Uniform	[36]
**Costs**	($AUD)	($AUD)		
Diagnosis (Non-Severe)	$58.48	$52.63-$64.33	Uniform	[37]
Diagnosis (Severe)	$29.24	$26.32-$32.16	Uniform	[37]
Diagnosis (Recurrent Inf)	$16.08	$14.48-$17.69	Uniform	[37]
Hospital (Non-Severe)	$800	$720-$880	Uniform	[38]
Hospital (Severe)	$3000	$2700-$3300	Uniform	[38]
Hospital (Recurrent Inf)	$1900	$1710-$2090	Uniform	[38]
Treatment (Non-Severe)	$3.71	$3.34-$4.08	Uniform	[29]
Treatment (Severe)	$47.43	$42.69-$52.17	Uniform	[29]
Treatment (Recurrent Inf)	$99.69	$89.72-$109.66	Uniform	[29]
**Transition Probabilities**		**(alpha; beta)**		
At-risk to:				
Remain at-risk	0.273	(236461; 629636)	Beta	[39]
Non-severe	0.0001	(93; 866004)	Beta	[39]
Severe	4.61E-06	(4; 866093)	Beta	[39]
Discharged healthy	0.725	(628408; 237689)	Beta	[39]
Dead	0.001	(1131; 864966)	Beta	[32]
**Non-severe infection to:**				
Remain non-severe	0.752	(70; 23)	Beta	[39]
Dead	0.000	(0.1; 93.1)	Beta	[39]
Discharged vulnerable 1	0.247	(23; 70)	Beta	[39]
**Severe infection to:**				
Remain severe	0.75	(3; 1)	Beta	[39]
Dead	0.000	(0.1; 4.1)	Beta	[39]
Discharged vulnerable 1	0.25	(1; 3)	Beta	[39]
**Discharged vulnerable 1 to:**				
Remain discharged vulnerable 1	0.829	(85; 632)	Beta	[39]
Censored	0.012	(1.3; 715.7)	Beta	[39]
Recurrent infection	0.110	(11.3; 705.7)	Beta	[39]
Dead	0.047	(4.9; 712.1)	Beta	[39]
**Discharged vulnerable 2 to:**				
Remain discharged vulnerable 2	0.846	(22.9; 166.1)	Beta	[39]
Censored	0.021	(0.6; 188.4)	Beta	[39]
Recurrent infection	0.126	(3.4; 185.6)	Beta	[39]
Dead	0.005	(0.1; 188.9)	Beta	[39]
**Recurrent infection to:**				
Remain recurrent infection	0.671	(19.3; 181.7)	Beta	[39]
Dead	0.059	(1.71; 199)	Beta	[39]
Discharged vulnerable 2	0.268	(7.7; 193.3)	Beta	[39]
**Discharged healthy to:**				
Remain discharged healthy	0.999	(847653; 88)	Beta	[39]
Dead	0.0001	(9.4; 623113)	Beta	[39]

The rate that patients move between compartments in the model was informed by a hospital morbidity dataset provided by the Western Australian Department of Health data linkage branch, which contained detailed demographic and clinical information for all admitted patients in the 18-months between 1 July 2011 and 31 December 2012. Those with an ICD-10 code for CDI (AO4.7) were extracted and examined in further detail. There were 844 *C*. *difficile* infections identified when non-severe (749) and severe (95) cases were combined. Patients who suffered an infection but did not require a stay in ICU were assumed to suffer ‘non-severe’ illness and patients who had an infection and a concurrent stay in ICU were classified as suffering ‘severe’ illness. Patients who experienced more than one hospitalisation due to the infection were tracked according to a non-identifiable patient code and these data informed the probability of recurrent infection. Patients whose cause of death included the ICD-10 code for *C*. *difficile* infection were used to inform the probability of dying from all of the infected health states. The probability of dying from the ‘discharged healthy’ state was estimated using data from Australian life tables, published by the AIHW [32]. Utility weights were assigned to all health states in the model, with weights reflecting a preference-based valuation of the health state, relevant to the patient’s infection status. Where possible, health utility weights were based on estimates from published studies. Expert opinion was used for those health states that could not be informed from the literature. The quality of the evidence used to populate the model was assessed according to published criteria described by Cooper et al [33]. The parameters included in the economic model are in Table 2.

The best available evidence was used in the study, but due to typical limitations in availability and access it was obtained from a range of sources, both within Australia and from published work outside the Australian setting.

### Estimating intervention effectiveness

The clinical effectiveness of each intervention was estimated from the results of a previously published, stochastic transmission model, which assessed the increase or decrease in *C*. *difficile* transmission as a result of that intervention [20]. Efficacy evidence was synthesised from six studies and was used to inform the effectiveness of both bundled approaches and stand-alone interventions. *Hygiene improvement* was found to have a large effect in decreasing disease incidence on its own (3.2 per 1000 bed days to 1.1 per 1000 bed days), but when coupled with another intervention, such as antimicrobial stewardship, little additional benefit was gained (2.7 per 1000 bed days to 2.3 per 1000 bed days). Meagre reduction in *C*. *difficile* incidence was observed for *Antimicrobial stewardship* (2.8 per 1000 bed days to 2.3 per 1000 bed days). *Fecal transplant* was found to be an ineffective control tool on its own (2.5 per 1000 bed days to 2.4 per 1000 bed days) and in combination with other transmission reduction strategies, such as *hygiene improvement*, there did not appear to be any collective effect (2.5 per 1000 bed days to 2.8 per 1000 bed days).

### Estimating cost-effectiveness

The cost-effectiveness of each intervention is shown by the incremental cost-effectiveness ratio (ICER), which is the change in costs divided by the change in health outcomes between the intervention and the comparator of standard care. Changes in health outcomes were estimated by combining information about intervention effectiveness and its subsequent impact on quality of life. Interventions that reduced *C*. *difficile* transmission compared with standard care had a flow-on reduction in the total number of infections, which resulted in a decreased number of patients who spent time in a state of reduced health. The total change in health outcomes was expressed in terms of quality-adjusted life years (QALYs). Changes in total costs were estimated by measuring total infection-related costs for standard care and each intervention. Infection-related costs are a combination of diagnosis, treatment and hospital bed-costs. The accrual of costs is linked to the number of infections that occur when one intervention is in place compared to another, and as seen in Table 2, differ according to infection severity due to disparities in treatment routine and hospital-bed costs. The incremental cost of running each intervention, compared with standard care, was also included when estimating the change to total costs.

Practical issues with using the ICER for decision-making exist, given that using the ratio of two numbers has awkward statistical properties [40]. In order to simplify this ratio information to a single number, the net monetary benefit (NMB) framework is used. Results are converted from the ICER to a net monetary benefit value, through the linear rearrangement of the ICER equation, as follows:

NMB=(WTPthresholdxChangeinEffects)–ChangeinCosts

The interpretation of cost-effectiveness becomes particularly simple using the NMB: a positive NMB indicates that a strategy is cost-effective and a negative NMB indicates that a strategy is not cost-effective. Using the net benefit framework gives decision-makers a clear comparison of multiple interventions as choosing the optimal intervention is as simple as selecting the intervention that provides the highest mean net monetary benefit. A willingness to pay of $42,000/QALY was used as the decision-making threshold for all analyses, which is in line with published estimates for cost-effectiveness in the Australian setting [41]. A lifetime time horizon has been used for this evaluation and as per previously published guidelines; future costs and health outcomes were both discounted at 5% per annum [42].

## Results

### Fixed value results

**Fig 2:**
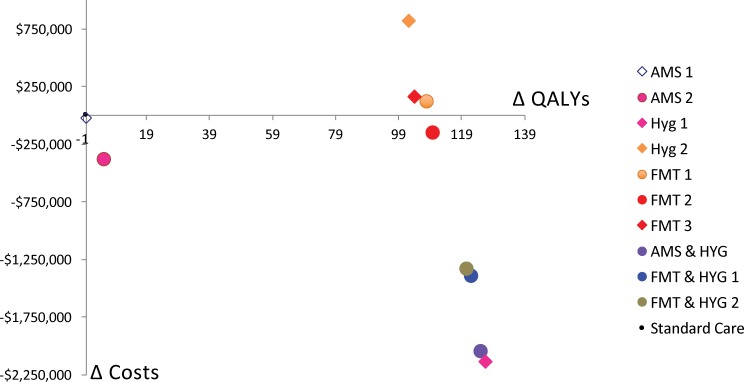
Incremental cost-effectiveness of interventions designed to reduce CDI.

Fixed value analysis does not account for any uncertainty in the decision. The results in Table 3 and Fig 2 show that of the ten interventions included, three were not cost-saving (HYG 2, FMT 1 & FMT 3). Hygiene improvement 1 (HYG 1) achieved the greatest health benefits (127 QALYs gained) and the lowest costs (over $2 million saved). Without consideration of uncertainty, HYG 1 dominated all other interventions because it has the greatest costs saved and health benefits gained.

**Table 3:** Incremental outcomes of all interventions compared to standard care.

Intervention	Incremental Outcomes		ICER
	**Costs**	**QALYs**	
**Standard Care**	-	-	Dominated
**AMS 1**	-$21,145	-0.09	Dominated
**AMS 2**	-$381,142	5.59	Dominated
**HYG 1**	**-$2,137,843**	**126.68**	**Cost-saving**
**HYG 2**	$818,790	102.11	Dominated
**FMT 1**	$119,595	107.87	Dominated
**FMT 2**	-$151,146	109.91	Dominated
**FMT 3**	$162,426	103.89	Dominated
**AMS & HYG**	-$2,052,003	125.14	Dominated
**HYG & FMT 1**	-$1,395,540	122.18	Dominated
**HYG & FMT 2**	-$1,332,211	120.73	Dominated

### Probabilistic results

**Fig 3:**
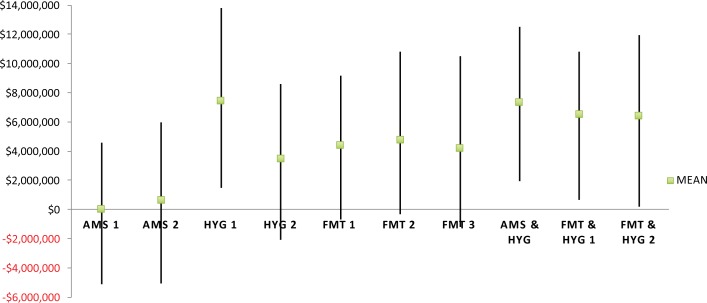
Net Monetary Benefits at a threshold of $42,000/QALY. Means and range of values for each intervention.

Parameter uncertainty was accounted for by using Monte Carlo simulation. In order to account for uncertainty in the model’s parameters, 10 000 simulations of the model were made, where in each replication a value randomly drawn from each parameter’s distribution was chosen. Table 2 shows the distributions that were applied to each of the model’s parameters. At a threshold level of $42,000 per QALY gained, HYG 1 provided the highest mean NMB ($7.5 million). The bundled approach of AMS/HYG delivered the next highest mean NMB ($7.3 million) and AMS 1 provided the lowest mean NMB of all interventions ($15,000). Fig 3 shows the results of the NMB analysis for all interventions. The intervention with the highest mean NMB is deemed to be the optimal decision and should be considered for adoption ahead of the other interventions [43].

### Scenario analysis

Uncertainties in other aspects of the evaluation also exist and are explored through scenario analyses. Different clinical scenarios were examined, where key parameters of the model were altered. The modified scenarios reflected plausible situations and test the robustness of the model. Four alternate scenarios were considered: (i) the method for valuing hospital-bed costs: where accounting costs for an ICU- and ward-bed were substituted by the healthcare decision-maker’s willingness to pay price, (ii) patient LOS was doubled, (iii) mortality rate was doubled to simulate serious outbreak conditions and, (iv) infection transmission rate was increased to simulate differences that may exist across the Australian setting. The results of scenario analyses are summarised in Table 4.

**Table 4:** Optimal intervention given different clinical scenarios ($42,000/QALY threshold).

Scenario	Intervention (Error probability)	Incremental Outcomes		Mean NMB (95% CI)
		*QALYs*	*Costs Saved*	
**1. WTP cost for bed days**	HYG 1 (0.74)	124.6	$535,723	$5,770,401 ($5,678,579–$5,862,224)
**2. Increased LOS**	HYG 1 (0.64)	123.8	$2,138,438	$7,337,386 ($7,238,532–$7,436,241)
**3. Increased infection rate**	AMS/HYG bundle (0.56)	164.6	$4,692,889	$11,605,010 ($11,480,838–$11,729,183)
**4. Increased mortality rate**	HYG 1 (0.64)	127.9	$2,026,338	$7,398,668 ($7,295,752–$7,501,586)

Hygiene improvement 1 (HYG 1) was optimal in most scenarios–when bed day cost was altered, LOS was increased and when mortality rate was increased. Only when the infection rate was increased was another intervention optimal. In this scenario the AMS/HYG bundled intervention provided the highest mean NMB. The scenario with the greatest certainty, indicated by error probability, was when the infection rate was increased. In this scenario the probability of incorrectly choosing the AMS/HYG bundle as optimal was 0.56. The error probabilities relating to the other scenarios were all high– 0.64 for scenarios 2 and 4 and 0.74 for scenario 1. This means that despite being the intervention that provided the highest NMB, the decision regarding HYG 1 as being optimal in multiple scenarios is highly uncertain.

## Discussion

Hospitals in Australia have access to multiple infection control interventions in the quest to reduce CDI. These results show the majority of interventions resulted in health improvements, indicated by QALYs gained. QALY gains were driven by a reduction in the number of infected patients as a result of a successful intervention, when compared with the number of infected patients who existed under the comparator. The results also showed that bundled interventions, as opposed to stand-alone interventions, offer the best opportunity for health improvements. Three out of the four highest-ranking interventions in terms of highest expected QALY gains were all bundles. The findings show that if decision-makers did not adopt any of the proposed interventions, improvements in health outcomes and cost savings would be forgone. If decisions are not made about the expenditure of scarce health resources, which includes decisions about disinvesting in initiatives that are not cost-effective, increased expenditure for no health outcome gain is probable.

The findings from model-based evaluations are always dependent on the assumptions made. Exclusions and simplifications are not uncommon limitations and this study was no different. There were limitations on how transition probabilities were estimated as well as limitations regarding the accuracy with which hospital-acquired and community-acquired infections are described due to cross-jurisdictional differences in data collection. The estimation of quality of life utility weights was not possible from primary data for all health states in the model and estimating costs is a difficult process, especially when they are not routinely collected at the hospital level for research purposes. Finally, the choice of interventions for inclusion in the project was deliberately simplified and did not include all possible *C*. *difficile-*related interventions. However, no economic evaluation of CDI has been undertaken in this setting, making the findings an important addition to existing clinical knowledge. Modelling studies also have noteworthy strengths–no prospective randomised control trial could be designed and commissioned to compare multiple interventions side-by-side, resulting in practical information for decision-makers. The majority of the data used to inform the results was derived from local sources, making the results very applicable to the Australian setting. Finally, the methods that have been used in this study are transparent, reproducible and follow published standards for this type of evaluation.

Prior to this research, Australian decision-makers have had limited access to economic evidence to inform their decision-making about managing risk of CDI. Although there is uncertainty in the results of this evaluation, doing nothing to change the management approach relating to *C*. *difficile* is a decision in itself that should be weighed up with appropriate consideration. Decision-makers need to understand that remaining with the status quo–simply maintaining one’s current or previous position—is itself an explicit decision. Put simply, if investments are not made in interventions that clearly yield gains in health outcomes compared to the status quo, the allocation and use of scarce healthcare resources is inappropriate.

### Limitations

Our study has limitations. The model does not account for epidemiologic parameters relating to CDI, such as the possibility of being admitted with infection. This is due to the issue that exists in the way that CDI is classified and reported in Australia. The definition of what constitutes an infection is well known and easily accessible [44], but there remain concerns about the accuracy with which hospital-acquired and community-acquired infections are described as there is bound to be some overlap between genuine hospital-acquired infections and community-acquired infections that are diagnosed and confirmed during a hospital stay. The absence of individual clinical test results meant that the classification of illness severity was simplified. For this project, it was assumed that the patients who had a confirmed case of infection and a parallel stay in ICU suffered *severe* infection and those who did not were classified as *non-severe*. This assumption may have resulted in an overestimation of severe cases, which could have had an effect on the results. Estimation of intervention efficacy was derived from the synthesis of a variety of evidence sources, which is typical for this type of evaluation. The particular estimates used in the model and presented here have been informed by the literature but are by no means definitive. As further evidence becomes available, the model can be updated and adapted to incorporate this.

Finally, given that the results were produced using predominantly Australian data, they are primarily intended for the Australian setting. However, the structure of the study is based on a flexible and adaptable Markov model, allowing inputs from newly identified literature or different healthcare settings to be included, making the results more appropriate for local settings in other jurisdictions around the world. This flexibility also extends to the capacity to include other interventions that exist to reduce *C*. *difficile* transmission in the hospital setting, if they are deemed more locally relevant than those presented here.

### Comparison to existing literature

There is little knowledge around the world about the cost of *C*. *difficile* from an economic perspective. Since 2014, there have been three economic evaluations published that focus on the cost-effectiveness of fecal microbiota transplant (FMT) in different settings [36;45–46]. All three studies found that FMT was a cost-effective approach to treating recurrent infection but none explored FMT as a method for inhibiting transmission of the pathogen. Nowak et al. undertook a study on the economic impact of an antimicrobial stewardship programme, but its focus was not solely on *Clostridium difficile* infection and included other HAIs–*vancomycin-resistant enterococci* (VRE) and *Methicillin-resistant Staphylococcus aureus* (MRSA) in the analysis [22]. The results are not specific to *C*. *difficile* and are not optimal for decision-making purposes. Nelson et al. conducted an economic evaluation of six different interventions in the USA-setting and found that implementing infection control bundles is more cost-effective than stand-alone interventions, which supports our findings [36].

## Conclusion

Until now, economic evidence relating to CDI has not been available for the Australian setting. The findings of this evaluation should be considered together with other relevant information that is appropriate at a local level, such as clinical outcomes, budget constraints and treatment priorities. The results support further investment in infection control interventions, providing evidence that such investment is an efficient use of scarce resources as cost-effective outcomes are likely to be realised.

## Supporting information

S1 File Further input parameters for economic model. (DOCX)

## References

[1] Kuijper E, Coignard B, & Tull P. Emergence of Clostridium difficile Associated Disease in North America and Europe. Clinical Microbiology and Infection, 2006 12(6): 2–18. 10.1111/j.1469-0691.2006.01580.x 16965399

[2] Van Gessel H, Riley TV, & McGregor A. Clostridium difficile Infection: An update for infection control practitioners. Healthcare Infection, 2009 14: 115–118.

[3] Lipp M, Nero DC, & Callahan MA. Impact of hospital acquired Clostridium difficile. Journal of Gastroenterology and Hepatology, 2012 27: 1733–1737. doi: 10.1111/j.1440-1746.2012.07242.x 22849881 10.1111/j.1440-1746.2012.07242.x

[4] Gravel D, Miller M, Simor A, Taylor G, Gardam M, McGeer A, et al Health care associated Clostridium difficile infection in adults admitted to acute care hospitals in Canada: A Canadian nosocomial infection surveillance program study. Clinical Infectious Diseases, 2009 48:568–576. doi: 10.1086/596703 19191641 10.1086/596703

[5] Barbut F & Petit JC. Epidemiology of *Clostridium difficile-*associated infections. Clinical Microbiology and Infection. 2001 7(8): 405–410 11591202 10.1046/j.1198-743x.2001.00289.x

[6] Kelly C, & LaMont JT. Clostridium difficile–more difficult than ever. New England Journal of Medicine, 2008 359:1932–1940. doi: 10.1056/NEJMra0707500 18971494 10.1056/NEJMra0707500

[7] Barbut F, Richard A, Hamadi K, Chomette V, Burghoffer B & Petit JC. Epidemiology of recurrences or reinfections of Clostridium difficile-associated diarrhea. Journal of Clinical Microbiology, 2000 38(6):2386–2388. 10835010 10.1093/gao/9781884446054.article.t031141 PMC86814

[8] van Nispen tot Pannerden CMF, Verbon A, & Kuipers EJ. Recurrent *Clostridium difficile* Infection. Drugs, 2011 71(7): 853–868. doi: 10.2165/11591230-000000000-00000 21568363 10.2165/11591230-000000000-00000

[9] Kwon JH, Olsen MA & Dubberke ER. The morbidity, mortality and costs associated with *Clostridium difficile* infection. Infectious disease clinics of North America. 2015 29(1): 123–134. doi: 10.1016/j.idc.2014.11.003 25677706 10.1016/j.idc.2014.11.003

[10] Hensgens M, Keessen EC, Squire MM, Riley TV, Koene MGJ, de Boer E, et al Clostridium difficile infection in the community: a zoonotic disease? Clinical Microbiology and Infection, 2012 18(7):635–645. doi: 10.1111/j.1469-0691.2012.03853.x 22536816 10.1111/j.1469-0691.2012.03853.x

[11] Cohen S, Gerding DN, Johnson S, Kelly CP, Loo VG, McDonald LC, et al Clinical Practice Guidelines for Clostridium difficile Infection in Adults: 2010 Update by the Society for Healthcare Epidemiology of America (SHEA) and the Infectious Diseases Society of America (IDSA). Infection Control and Hospital Epidemiology, 2010 31(5). 10.1086/651706 20307191

[12] Graves N. Economics and preventing hospital-acquired infection. Emerging Infectious Diseases, 2004 10(4):561–6. doi: 10.3201/eid1004.020754 15200842 10.3201/eid1004.020754 PMC3086182

[13] Graves N, Halton K, Lairson D. Economics and preventing hospital-acquired infection: broadening the perspective. Infection Control & Hospital Epidemiology. 2007 28(2):178–84. 17265399 10.1086/510787

[14] Gray AM, Clarke PM, Wolstenholme JL & Wordsworth S. Applied methods of cost-effectiveness analysis in health care. Oxford: Oxford University Press 2011.

[15] Yakob L, Riley TV, Paterson DL, & Clements ACA. Clostridium difficile exposure as an insidious source of infection in healthcare settings: an epidemiological model. BMC Infectious Diseases, 2013 13. 10.1186/1471-2334-13-376 PMC3751620 23947736

[16] MacDougall C & Polk R. Variability in rates of Use of antibacterials among 130 US hospitals and risk‒adjustment models for interhospital comparison. Infection Control & Hospital Epidemiology. 2008 29: 203–211. 18257689 10.1086/528810

[17] Polk R, Fox C, Mahoney A & Letcavage JCM. Measurement of adult antibacterial drug Use in 130 US hospitals: comparison of defined daily dose and days of therapy. Clinical Infectious Diseases. 2007, 44: 664–670. doi: 10.1086/511640 17278056 10.1086/511640

[18] Rafii F, Sutherland J & Cerniglia C. Effects of treatment with antimicrobial agents on the human colonic microflora. Ther Clin Risk Manag. 2008, 4: 1343–1358. 19337440 10.2147/tcrm.s4328 PMC2643114

[19] OECD: Health at a Glance 2011: OECD Indicators. 2011, OECD Publishing, doi: 10.1787/health_glance-2011-en

[20] Yakob L, Riley TV, Paterson DL, & Clements ACA. Assessing control bundles for Clostridium difficile: a review and mathematical model. Emerging Microbes and Infection, 2014 3. 10.1038/emi.2014.43 PMC4078791 26038744

[21] Ryan K, Russo PL, Havers S, Heard K, Bellis K & Grayson ML. Development of a standardized approach to observing hand hygiene compliance in Australia. Healthcare Infection, 2012 17:115–121.

[22] Nowak M, Nelson RE, Breidenbach JL, Thompson PA, & Carson PJ. Clinical and economic outcomes of a prospective antimicrobial stewardship program. American Journal of Health System Pharmacy, 2012. 10.2146/ajhp110603 22899745

[23] Climo M, Israel DS, Wong ES, Williams D, Coudron P, & Markowitz SM. Hospital wide restriction of clindamycin: effect on the incidence of Clostridium difficile-associated diarrhea and cost. Annals of Medicine, 1998 128:989–995. 10.7326/0003-4819-128-12_part_1-199806150-00005 9625685

[24] Akpan MR, Ahmad R, Shebl NA & Ashira-Oredope D. A review of quality measures for assessing the impact of antimicrobial stewardship programs in hospitals. Antibiotics, 2016 5(1). 10.3390/antibiotics5010005 PMC4810407 27025520

[25] Talpaert MJ, Rao GG, Cooper BS & Wade P. Impact of guidelines and enhanced antibiotic stewardship on reducing broad-spectrum antibiotic usage and its effect on incidence of *Clostridium difficile* infection. Journal of Antimicrobial Chemotherapy, 2011 66: 2168–2174. doi: 10.1093/jac/dkr253 21676904 10.1093/jac/dkr253

[26] Avadhani A, & Miley H. Probiotics for prevention of antibiotic-associated diarrhoea and Clostridium difficile-associated disease in hospitalised adults–A meta analysis. Journal of the American Academy of Nurse Practitioners, 2011 23:269–274. doi: 10.1111/j.1745-7599.2011.00617.x 21649768 10.1111/j.1745-7599.2011.00617.x

[27] Page K, Graves N, Halton K, & Barnett AG. Humans, ‘things’ and space: costing hospital infection control interventions. Journal of Hospital Infection, 2013 84:200–205. doi: 10.1016/j.jhin.2013.03.006 23688708 10.1016/j.jhin.2013.03.006

[28] Frick K. Micro-Costing Quantity Data Collection Methods. Medical Care. 2009 47: S76–S81. doi: 10.1097/MLR.0b013e31819bc064 19536026 10.1097/MLR.0b013e31819bc064 PMC2714580

[29] Pharmaceutical Benefits Scheme. PBS Online. 2015; Available from: www.pbs.gov.au

[30] Turnidge J, Kotsanas D, Munckhof W, Roberts S, & Bennett C. Staphylococcus aureus bacteraemia: a major cause of mortality in Australia and New Zealand. Medical Journal of Australia, 2009 191(7):368–373. 19807625 10.5694/j.1326-5377.2009.tb02841.x

[31] Stewardson A, Harbarth S, & Graves N. Valuation of Hospital Bed-Days Released by Infection Control Programs: A Comparison of Methods. Infection Control and Hospital Epidemiology, 2014 35(10):1294–1297. doi: 10.1086/678063 25203185 10.1086/678063

[32] Australian Bureau of Statistics. 2015. Available from: http://www.abs.gov.au/AUSSTATS/abs@.nsf/DetailsPage/3302.0.55.0012011-2013?OpenDocument

[33] Cooper N, Coyle D, Abrams K, Mugford M & Sutton A. Use of evidence in decision models: an appraisal of health technology assessments in the UK since 1997. Journal of Health Services Research and Policy, 2005 10(4):245–250. doi: 10.1258/135581905774414187 16259692 10.1258/135581905774414187

[34] Konijeti G, Sauk J, Shrime MG, & Ananthakrishnan AN. Cost-effectiveness of competing strategies for management of recurrent Clostridium difficile infection: A decision analysis. Clinical Infectious Diseases, 2014 58(11):1507–1514. doi: 10.1093/cid/ciu128 24692533 10.1093/cid/ciu128 PMC4017891

[35] Riley, T., Personal communication, 2014.

[36] Nelson RE, Jones M, Leecaster M, Samore MH, Ray W, Huttner A, et al An Economic Analysis of Strategies to Control Clostridium difficile Transmission and Infection Using an Agent-Based Simulation Model. PLoS ONE, 2016 11(3). 10.1371/journal.pone.0152248 PMC4816545 27031464

[37] Medicare Benefits Schedule. MBS Online. 2015; Available from: www.mbsonline.gov.au.

[38] Rechner I, & Lipman J. The costs of caring for patients in a tertiary referral Australian intensive care unit. Anaesthesia and Intensive Care, 2005 33(4):477–482. 16119489 10.1177/0310057X0503300409

[39] Western Australia Department of Health (Data Linkage Branch), dataset 201306.04, 2014.

[40] Stinnett A, & Mullahy J. Net health benefits: a new framework for the analysis of uncertainty in cost-effectiveness analysis. Medical Decision Making, 1998 18(S68–S80). doi: 10.1177/0272989X98018002S09 9566468 10.1177/0272989X98018002S09

[41] George B, Harris A, & Mitchell A. Cost-effectiveness analysis and the consistency of decision-making: evidence from pharmaceutical reimbursement in Australia (1991 to 1996). Pharmacoeconomics. 2001 19:1103–1109. 11735677 10.2165/00019053-200119110-00004

[42] Gravelle H, Brouwer W, Niessen L, Postma L & Rutten F. Discounting in economic evaluations: stepping forward towards optimal decision rules. Health Economics, 2007 16: 307–317. doi: 10.1002/hec.1168 17006970 10.1002/hec.1168

[43] Barton G, Briggs AH, & Fenwick EAL. Optimal cost-effectiveness decisions: the role of the cost-effectiveness acceptability curve (CEAC), the cost-effectiveness acceptability frontier (CEAF) and the expected value of perfect information (EVPI). Value in Health, 2008 11(5). 10.1111/j.1524-4733.2008.00358.x 18489513

[44] Cheng A, Ferguson JK, Richards MJ, Robson JM, Gilbert GL, McGregor A, et al Australasian society for infectious diseases guidelines for the diagnosis and treatment of Clostridium difficile infection. Medical Journal of Australia, 2011 194:353–358. 21470086 10.5694/j.1326-5377.2011.tb03006.x

[45] Merlo G, Graves N, Brain D & Connelly L. Economic evaluation of fecal microbiota transplantation for the treatment of recurrent Clostridium difficile infection in Australia. Journal of Gastroenterology and Hepatology, 2016. 10.1111/jgh.13402 27043242

[46] LaPointe-Shaw L, Tran KL, Coyte PC, Hancock-Howard RL, Powis J, Poutanen SM, et al Cost-Effectiveness Analysis of Six Strategies to Treat Recurrent Clostridium difficile Infection. PLoS ONE, 2016 11(2). 10.1371/journal.pone.0149521 PMC4769325 26901316

