# Supplementary material for: Economic evaluation of interventions designed to reduce Clostridium difficile infection
Source: PLoS One. 2018 Jan 3;13(1):e0190093. doi: 10.1371/journal.pone.0190093 (PMC5752026; doi:10.1371/journal.pone.0190093)
Supplement: S1 File — (DOCX) [file pone.0190093.s001.docx]

**Further input parameters for economic model**

This information should be read in conjunction with table 2 from the manuscript titled ‘input variables for the Markov model’. All information required to replicate the results of the study is included in the manuscript and in this supporting file.

Table S1: Further input parameters for economic model

| Parameter | Fixed Value | Range | Distribution |
| --- | --- | --- | --- |
| Length of stay | ***Days*** | | |
| Non-Severe | 2.2 | (1.54 to 2.86) | Uniform |
| Severe | 4.4 | (3.08 to 5.72) | Uniform |
| Recurrent infection | 8 | (5.60 to 10.40) | Uniform |
| Intervention costs | ***Incremental costs from standard care (per annum)*** | | |
| Hygiene Improvement | $146,988 | ($132,289 to $161,687) | Uniform |
| AMS | $240,388 | ($216,349 to $264,427) | Uniform |
| FMT | $$89,066 | ($80,159 to $97,972) | Uniform |
| AMS & HYG bundle | $387,377 | ($348,639 to $426,114) | Uniform |
| FMT & HYG bundle | $236,054 | ($212,449 to $259,659) | Uniform |
| Cohort | N = 860,000 |  |  |
